# Nanoscale mapping of plasmon and exciton in ZnO tetrapods coupled with Au nanoparticles

**DOI:** 10.1038/srep19168

**Published:** 2016-01-12

**Authors:** Giovanni Bertoni, Filippo Fabbri, Marco Villani, Laura Lazzarini, Stuart Turner, Gustaaf Van Tendeloo, Davide Calestani, Silvija Gradečak, Andrea Zappettini, Giancarlo Salviati

**Affiliations:** 1 CNR-IMEM, Parco Area delle Scienze 37/A, IT 43124 Parma, Italy; 2 EMAT, University of Antwerp, Groenenborgerlaan 171, BE 2020 Antwerp, Belgium; 3 Department of Materials Science and Engineering, Massachusetts Institute of Technology, Cambridge, Massachusetts (USA)

## Abstract

Metallic nanoparticles can be used to enhance optical absorption or emission in semiconductors, thanks to a strong interaction of collective excitations of free charges (plasmons) with electromagnetic fields. Herein we present direct imaging at the nanoscale of plasmon-exciton coupling in Au/ZnO nanostructures by combining scanning transmission electron energy loss and cathodoluminescence spectroscopy and mapping. The Au nanoparticles (~30 nm in diameter) are grown *in-situ* on ZnO nanotetrapods by means of a photochemical process without the need of binding agents or capping molecules, resulting in clean interfaces. Interestingly, the Au plasmon resonance is localized at the Au/vacuum interface, rather than presenting an isotropic distribution around the nanoparticle. On the contrary, a localization of the ZnO signal has been observed inside the Au nanoparticle, as also confirmed by numerical simulations.

The combination of a mature semiconductor nanostructure platform with newborn nano- plasmonics technology paves the way to novel technological applications. That strongly leverages confined optical fields to manipulate the optical responses (*i.e.*, scattering, absorption, and emission) of semiconductor structures at the nanoscale. It has recently been shown that the electromagnetic plasmonic-photonic coupling in metal/semiconductor nanostructures may enable the development of several device types. For instance, the surface plasmon resonance of Ag metallic nanoparticles (NPs) has been used to increase light scattering and absorption in silicon nanowires based hybrid solar cells, enhancing the device efficiency^1^. Furthermore, the optical emission of InGaN LEDs has been enhanced through the coupling with a nanostructured metal film: a significant improvement of the internal quantum efficiency has been measured when Ag or Al layers were deposited 10 nm above an InGaN light-emitting layer^2^. In addition, recent work demonstrated that when GaAs NWs are coupled with gold nano-antennas, they display interesting non-linear optical properties because of the resulting electromagnetic coupling^3^. These findings represent an initial step toward the development of innovative metal−semiconductor resonant nanostructures for the realization of next generation devices.

In the following we focus on metallic nanoparticles since they are known to show size- tunable plasmon absorption by electrons confined to dimensions smaller than the electron mean free path^4^. NPs can easily be conjugated to semiconducting nanocrystals (*e.g.*, Si, ZnO, TiO_2_ …) to enhance photo catalytic behavior and band edge emission of the semiconductor^5^,^6^. This was demonstrated on Ag-ZnO coupled nanospheres^7^ due to the strong Ag plasmon resonance at ∼3.3 eV. The modification of the electronic properties of nanostructured interfaces between Ag and ZnO has been attributed to an electromagnetic coupling between excitons and plasmons^8^. Even photoluminescence and Raman scattering are enhanced by the coupling, and it was demonstrated in thin Au NPs covering ZnO films^9^,^10^, while an enhancements of UV photo-detection^11^ as well as of photovoltaics and photochemical reaction^12^,^13^ were demonstrated in other Au-ZnO nanocomposites. All these results can be considered as indirect proofs of an exciton-plasmon coupling.

On the other hand, recent advances in scanning transmission electron microscopy (STEM), combined with electron energy loss spectroscopy (EELS), enable direct spectroscopy and imaging of plasmon resonances at a combined high spatial (<1 nm) and energy resolution (<0.15 eV). EELS allows direct mapping of resonant modes as surface plasmon polaritons in thin films or localized plasmon resonances (LPR) in individual metallic NPs^14^,^15^. This can be done thanks to: i) probe aberration correction that boosts the signal in the spectrum by using larger illumination angles; ii) cold field emitters or monochromators that enhance the energy resolution and reduce the high tail of the elastically transmitted electrons, which otherwise dominate the acquired spectrum in the IR-vis frequency range.

EELS investigations can be complemented by STEM-Cathodoluminescence (CL) spectroscopy and imaging to study the optical emission. As a result both high energy and high spatial resolution maps can be obtained^16^,^17^,^18^,^19^, to get information on the excitation and radiative de-excitation of the metal/semiconductor system. However, most of EELS maps on nanoparticles in literature are acquired with the aim to image the LPR modes in metallic NPs, or the near band edge onset (NBE) in semiconducting nanocrystals as a function of their size or geometry^17^,^18^,^19^,^20^,^21^,^22^,^23^,^24^,^25^, while intentionally avoiding the effect of interfaces.

In the present work, we provide an experimental visualization of the effect of electromagnetic coupling on the spatial localization of excitons from ZnO nanotetrapods (TPs) and of the plasmon resonance from Au NPs by aberration corrected STEM-EELS imaging, STEM-CL-spectroscopy and mapping, and numeric simulations in the Mie framework^18^ The effect of interaction can be demonstrated not only by an energy transfer from the ZnO NBE to the Au LPR as can be inferred from CL, but also by the spatial extension of the ZnO excitations (NBE and excitons) into the Au particles as seen in EELS. The present TPs offer an advantage with respect to other varieties of ZnO nanostructures, due to a negligible intensity of “green” emission arising from the ZnO surface defects in the visible band (see Supplementary Fig. S1 online). This enables the detection of the Au plasmon resonance in the nano-composite material without misleading contributions. Similar plasmon-exciton nanostructures, where electromagnetic coupling can be tuned both in energy and space, may become key blocks in future low consumption solar cells, optical modulators, and biodetectors at the nanoscale.

## Results

### Synthesis of the Au/ZnO nanostructures

**Figure 1:**
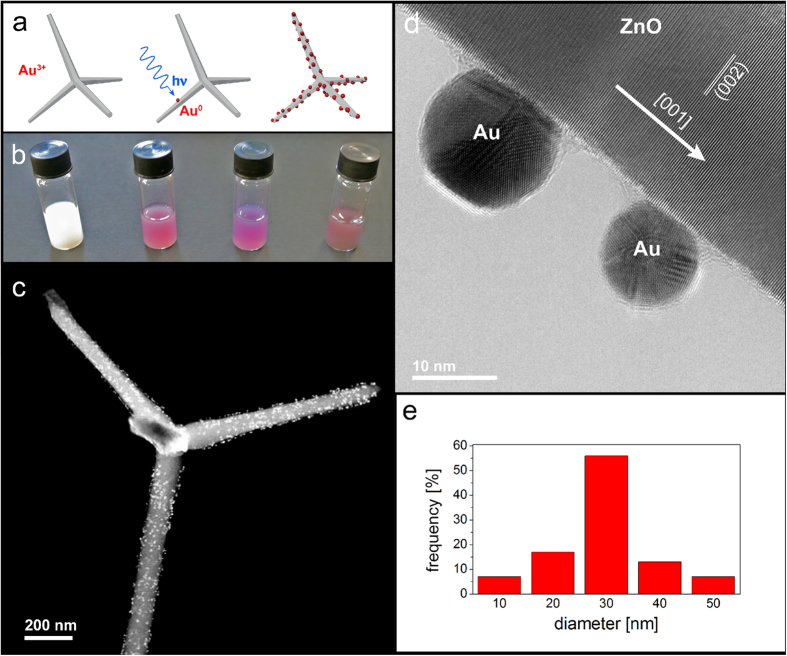
Au functionalization of ZnO TP. (**a**) Sketch of ZnO TP functionalization with Au NPs by means of photo-chemical reaction. A ZnO TP dispersion is irradiated with UV light and photo-generated carriers promote *in-situ* Au(III) to Au(0) reduction. (**b**) Au NPs dimensions can be tailored by changing the overall quantity of Au precursor and illumination time. (**c**) A representative Au NPs/ZnO TP as seen in HAADF-STEM. (**d**) HRTEM image of two Au NPs in close contact with the ZnO arm surface. (**e**) Size distribution of the grown Au NPs on ZnO TPs.

The Au particles were synthesized *in-situ* on ZnO tetrapods^20^, by means of photo-reduction of a gold precursor (HAuCl_4_), without the need of any other reagent (Fig. 1) (details in the methods section). Previous investigations reporting the synthesis of noble metal nanostructures by photoreaction, employed external reducing agents or capping molecules^21^,^22^,^23^,^24^,^25^,^26^, or further relied on post annealing^11^, to pledge chemical reduction, ensure nanoparticles shape control, and avoid aggregation. In the present study, instead, we provide a straightforward protocol to tailor the overall dimension of the as-synthesized Au NPs, by adjusting the total quantity of the gold precursor and the irradiation time, while the Au NPs density on the ZnO surface depends mostly on the concentration of the tetrachloroauric solution. No thermal post-annealing was performed. As a result, gold nanoparticles with an average diameter of 30 nm were grown on the ZnO TPs surface.

HRTEM images confirm the Au NPs are in contact with the ZnO pod and present a flat Au/ZnO interface (Fig. 1d), demonstrating that the Au particles heterogeneously nucleate on the ZnO surface. No contamination from the solvent is visible on the NP surface.

The main advantage of this synthetic procedure is that the Au/ZnO interface is clean and no contamination arising from organic molecules or post growth annealing are present. As a consequence the imaging of the spatial localization of the plasmon-exciton coupling is straightforward.

### Radiative excitation and de-excitation of the metal/semiconductor system

**Figure 2:**
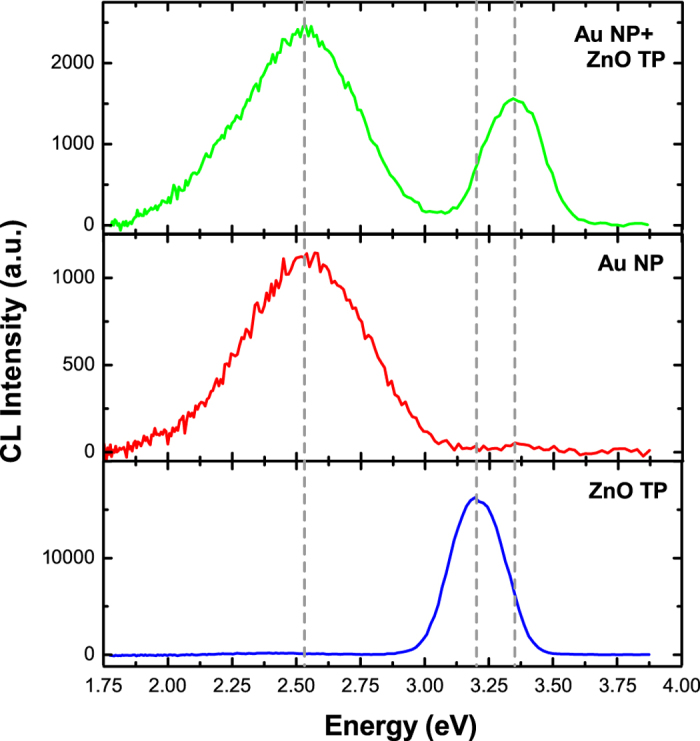
Cathodoluminescence spectra of the Au/ZnO nanostructure. CL spectra from the as-synthesized ZnO TPs (blue curve), a pure Au NPs reference sample (red curve), and the Au NPs grown on the ZnO TPs (green curve).

Figure 2 shows STEM-CL spectra of bare ZnO TPs (blue curve), Au NPs (red curve) and the Au-NPs/ZnO-TP nanosystem (green curve). The ZnO TPs spectrum shows a band at 3.3 eV related to the ZnO near band edge (NBE)^27^, while the gold nanoparticle spectrum shows a band at 2.4 eV, corresponding to the Au LPR^14^. The nanosystem spectrum presents both a two-fold enhanced Au LPR emission and a significant quenched ZnO NBE emission, approximately by a factor of ten. Note that by acquiring the CL spectra over a whole arm of the tetrapod, the Au NPs polydispersity (20 nm < *d* < 40 nm, see histogram in Fig. 1e) induces a broadening of the LPR emission. In addition, the NBE emission is blue-shifted by 130 meV. Previous work on the optical properties of Au-NPs/ZnO nanosystems presents contradicting results. For instance, an up to seven-fold increase of the ZnO NBE emission after Au NPs decoration has been reported, explained by a surface defect passivation or by excited electrons in the Au NPs that tunnel into the conduction band of ZnO mediated by ZnO intra-gap states^28^,^29^,^30^. Meanwhile, a quenching of the ZnO NBE emission after decoration with Au-NPs was also found, likely caused by excited electron transfer from the ZnO conduction band to the Au NPs^31^. Since we did not detect any intra-gap states related emissions in as synthesized ZnO tetrapods (Fig. 2b), the ZnO NBE enhancement mediated by intra-gap states cannot occur. The enhancement of the Au LPR emission and the concurrent quenching of the ZnO NBE emission demonstrates the occurrence of an energy transfer from the ZnO to the Au NPs. The decrease of the ZnO NBE emission can be due both to an electronic and/or to an optical effect *i.e.*, the electrons generated in the ZnO can tunnel to the Au NPs, and/or the ZnO NBE emission can be absorbed by the Au nanoparticles interband transitions (see Supplementary Fig. S2 online). As a result-in both cases-the radiative emission of the Au NP LPR is enhanced.

The ZnO NBE blue-shift suggests the existence of a direct electromagnetic interaction between the Au NPs and the ZnO nanostructure. In particular, the blue-shift of the Au NP decorated ZnO NBE emission was previously attributed to a decrease in the lifetime of the ZnO excitons, not permitting all of them to reach potential minima^32^. Spot-mode CL spectra, taken on a single Au NP, detected both the Au LPR emission, as expected, and the ZnO NBE emission, confirming the interaction (see Supplementary Fig. S3c online).

### High resolution spatial localization of plasmon-exciton coupling

**Figure 3:**
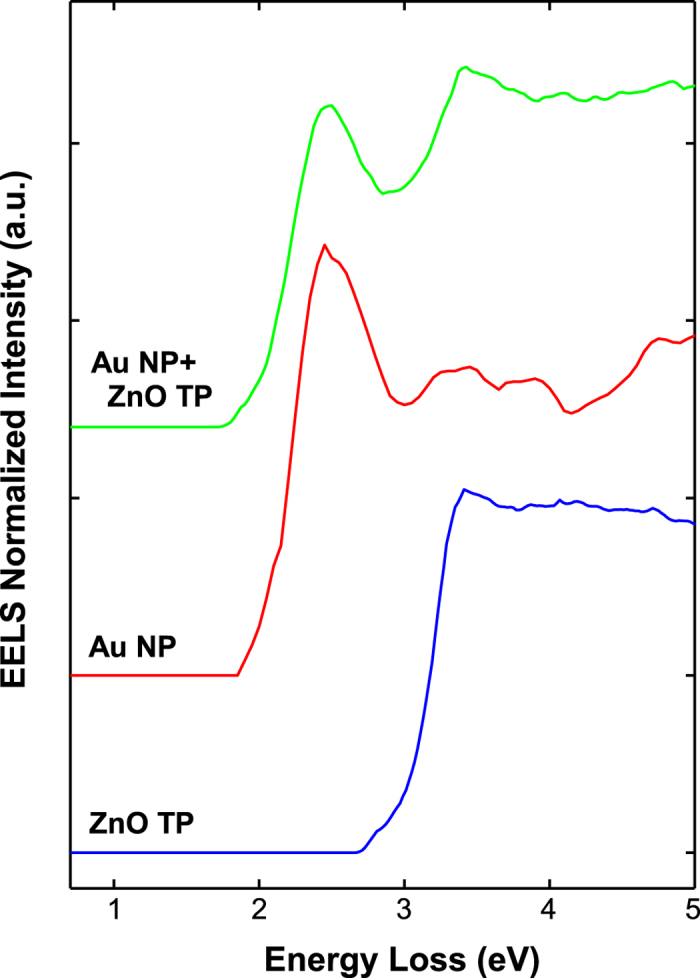
Energy loss spectra of the Au/ZnO nanostructure. EEL spectrum from a single Au NP on ZnO TP (green curve) and the two reference spectra from an isolated Au NP (red curve) and pristine ZnO (blue curve) used in the NLLS fitting. The spectra were normalized (integral area) in the interval 1–5 eV.

EELS cannot provide a direct measure of the energy transfer in the Au/ZnO nanostructures as for CL, but can give a proof of the coupling by studying the spatial distribution of the Au plasmon (LPR) and of the ZnO excitations (NBE and excitons). For this reason, we performed high-resolution EELS mapping on a FEI Titan microscope equipped with a CEOS probe corrector and an electron monochromator. A representative spectrum close to the surface (*i.e.*, taken in the so-called ‘aloof’ position) from a single Au NP, is shown in Fig. 3 (green line), and presents the typical LPR of Au at 2.4 eV^33^,^34^ together with a feature ascribed to the ZnO NBE at 3.3 eV^35^. However, the Au NP presents interband transitions involving *d* and *sp* orbitals^36^ that overlap with the ZnO NBE (as confirmed by absorption spectroscopy reported in Supplementary Fig. S2 online). To subtract the contribution from Au interband transitions, we performed a nonlinear fitting using two reference spectra (see the Methods section for further details). An EEL spectrum from a single Au particle on holey-carbon thin film (best approximating an isolated particle) was used to describe the LPR component including the interband transitions (red line in Fig. 3), while the ZnO NBE component is obtained from an EEL spectrum acquired close to the surface of a bare ZnO tetrapod (blue line in Fig. 3). An example of resulting fitting curve is shown in Supplementary Fig. S5 online. The EELS fits agree with a negligible red-shift of the Au LPR and a small blue-shift of the ZnO band edge (100 ± 20 meV).

## Discussion

**Figure 4:**
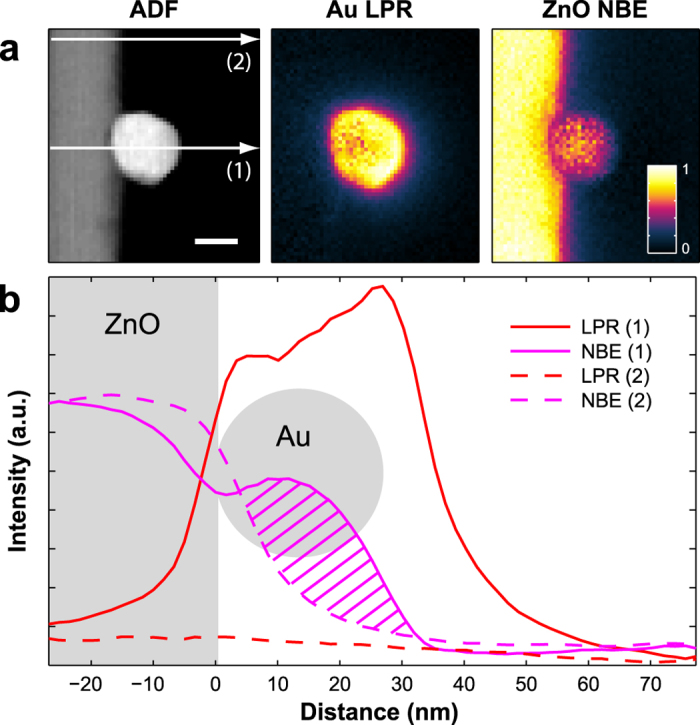
Evidence of coupling from energy loss maps. (**a**) HAADF image of the Au/ZnO interface (scale bar 20 nm), together with the Au plasmon (LPR) component and the ZnO near band edge (NBE) component after fitting of the reference spectra. The LPR is localized towards the Au/vacuum side, while a signal resembling the ZnO NBE extends clearly inside the Au NP, as confirmed by the shaded region in the integrated linescans in (**b**).

Figure 4 compares the HAADF image with the spatial distributions of the LPR and NBE components resulting from the fitting. Interestingly, the LPR component is focused on the vacuum side surface of the Au particle, whereas it reduced at the ZnO interface. On the other hand, the NBE component arising from the ZnO TP, extends across the Au NP. This feature cannot be explained by considering the delocalization of the inelastic scattering into the vacuum region; the signal extends further on the particle rather than on the pod surface. This is clearly observed in the plot of Fig. 4b, obtained from a 5 pixels wide integration (linescans) from a path crossing the particle (1), and from a path away from the particle (2). If, on the contrary, the intensity of the signal at 3.3 eV would belong to Au interband transitions, the Au NP particle should be seen as transparent in the NBE map.

**Figure 5:**
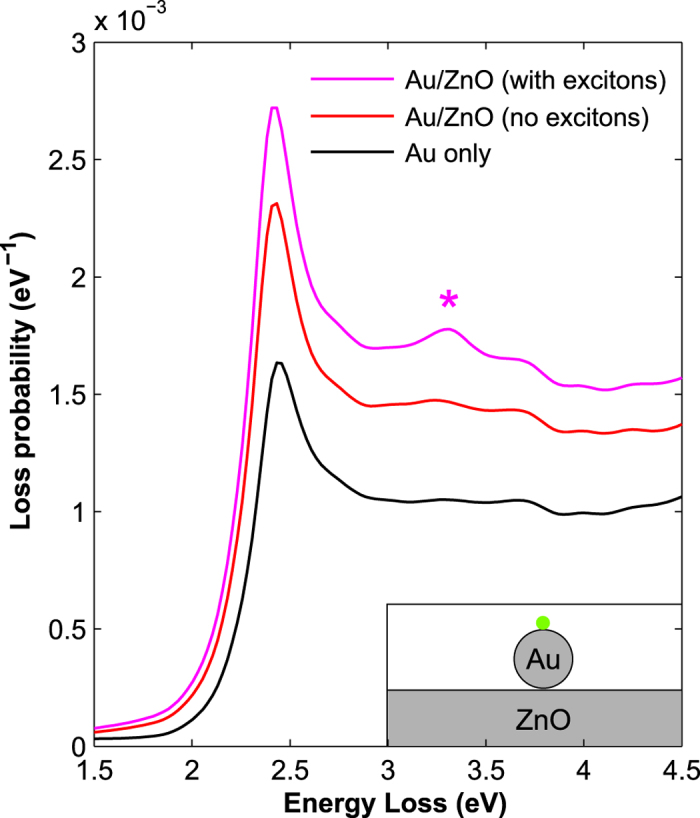
Simulation of the energy loss spectra. Simulated spectra at the ‘aloof’ position (green dot in the sketch) for the Au NP on ZnO system. A simulation with an experimental dielectric function (magenta curve) is compared to the simulation with a DFT function (red curve), which neglects excitonic transitions. The delocalized signal from the ZnO TP was subtracted from both spectra. The spectrum from an isolated Au particle (black curve) is shown for comparison. The dielectric function with the excitonic transitions gives a higher contribution on the particle at 3.3 eV (*).

To further verify this assumption, we simulated the EEL spectra using the boundary element method MNPBEM^37^ for an Au spherical particle on a cylindrical ZnO pod. The simulated spectra at the outer surface of the Au NP (dot green mark) are presented in Fig. 5, which compares results obtained using an experimental dielectric function^38^ and a DFT-based dielectric function^39^. The former includes excitonic transitions, which are neglected in the latter. The delocalized signal from the ZnO TP has been subtracted from both spectra by performing a simulation without the Au NP. The spectrum from an isolated Au NP (red curve) is shown for comparison. The dielectric function with the excitonic transitions gives a further contribution at 3.3 eV on the particle that adds to the NBE onset contribution (see the feature marked by the asterisk in Fig. 4). A comparison between the experimental and simulated signals (as derived from linescans integration from the 2.4 eV and 3.3 eV raw maps) is shown in Supplementary Fig. S4 online. A further simulation using a SiO_2_ substrate (which has negligible absorption at 3.3 eV) does not show any extra feature in this energy range (see Supplementary Fig. S6 online).

We have provided a direct imaging of plasmon and exciton signals and the effect of their interaction even if they are well separated in energy, making use of ZnO nanotetrapods decorated with Au nanoparticles as a prototype system. The effect of coupling is confirmed by an enhancement of the Au LPR intensity, and by the decrease and blue-shift (0.13 eV) of the ZnO TPs NBE emission, as found by STEM-CL spectroscopy. A further evidence is given by spatially resolved STEM-EELS mapping with probe aberration correction showing that the Au nanoparticles act as lenses for the ZnO excitations which spatially enter the Au NPs. As a consequence, the Au localized plasmon resonance is confined toward the nanoparticles/vacuum interface, as confirmed by the simulations. The absence of organic ligands on Au NPs and interdiffusion of Zn or Au at the interface, are key aspects to prove an efficient coupling in similar nanosystems. The LPR confinement toward the outer region of the interface may be used to improve the sensitivity of surface enhanced Raman spectroscopy, to investigate a variety of adsorbed molecular systems on metal nanoparticles^40^. Further, thanks to the biocompatibility of ZnO^41^ and the absorption of the NPs in the visible range further increased in the near UV, this would enable significant insights into the development of novel metal-semiconductor architectures for biomedicine, optoelectronics, and (photo)catalytic applications.

## Methods

### Preparation of Au nanoparticles decorated ZnO nanotetrapods

10 mg of ZnO TPs were dispersed in 50 ml of isopropyl alcohol (IPA) by sonication. The suspension was illuminated with a 300 W halogen lamp, and 100 uL from a 1 mM of a HAuCl_4_ aqueous solution were gradually added under vigorous stirring every 10 minutes for 2 hours. The nanostructures were then washed with deionized water, and collected by centrifugation. Upon illumination with photon energy greater than the ZnO band gap, electron-hole pairs are generated inside the semiconductor and act as a driving force for the redox process. Conduction band electrons promote the reduction of Au^3+^ cations chemisorbed at the semiconductor surface, giving rise to the heterogeneous nucleation of Au NPs on the ZnO surface. The electro-neutrality is retained because of IPA oxidation to acetone by the photo-generated holes.

### Cathodoluminescence spectroscopy

STEM-CL measurements were carried out on a JEOL JEM-2011 equipped with a commercial Gatan MonoCL3 system at an accelerating voltage of 120 keV and a spot size of 1.5 nm^42^. The STEM-CL spectra of different samples under analysis were acquired at the same magnification (200 k) and beam current (3 nA). The reference spectrum for Au NPs was acquired from a sample deposited on a holey TEM grid with a similar amount of nanoparticles as the Au NPs decorated ZnO sample. The ZnO tetrapod spectra, shown in Fig. 2b are representative of a cathodoluminescence statistical study. More than twenty tetrapods, with a comparable size (as arm diameter), were studied at the same magnification and beam parameters for each sample. The maximum variation of the CL integrated intensities is 3% in the case of the ZnO NBE emission and 10% in the case of the Au NPs LPR. Where not indicated, the CL spectra have to be considered as integrated in the scanning area. Montecarlo simulations (see Supplementary Fig. S7 online) are carried out in order to evaluate the electron excitation in the case of bare ZnO and Au NPs decorated ZnO.

### Electron energy loss spectroscopy

EEL spectrum images, or (*x, y, E*) datasets, were acquired in high angle annular dark field (HAADF) mode on a FEI Titan ‘cubed’ microscope equipped with a CEOS probe corrector, an electron monochromator, and a Gatan Enfinium spectrometer, giving an energy resolution in the datasets of Δ*E* = 0.15 eV (as measured from the FWHM of the elastic peak). The spectra were acquired on a CCD camera at a dispersion of 0.05 eV/pixel. The acquisition conditions were *E* = 120 keV, α = 30 mrad, and β = 60 mrad with the choices of condenser and spectrometer apertures. To subtract the effect of the elastic contribution each spectrum at pixel (*x, y*) was normalized to its integral in *E* (to take account approximately of higher energy losses, the inelastic part is extended from 50 eV to 200 eV with a linear decreasing signal). In this way we recover the same amounts of electrons in each spectrum at each pixel (*i.e.*, the spectrometer collects always all the scattered electrons). To approximately separate the two contributions originating from the LPR plasmon and the ZnO band edge, respectively, we used Levenberg–Marquardt (LM) fitting for Poisson statistics as implemented in EELSMODEL^43^, using three components: i) a power-law background (mimicking the tail of the (quasi) zero-loss peak; ii) a reference spectrum for the LPR from a weakly interacting Au particle (considering also the inter-band transitions); iii) a ZnO reference spectrum acquired from a pure ZnO TP.

### Simulations

EEL spectra were calculated with the NMPBEM eels package^37^, using the full *bem* (boundary element method) solver. Both Au and ZnO dielectric function were determined from (*n, k*) tabulated indices from literature. The complex refractive index measured experimentally (with the excitons) was taken from Isashi *et al.*^38^, while the one without the exciton contribution was taken from the DFT calculation in Calzolari *et al.*^39^. For Au we used the internal reference of the NMPBEM code. The diameter of the Au particle and the ZnO pod in the simulation are 25 nm and 80 nm, respectively, to match experimental values.

## Additional Information

**How to cite this article**: Bertoni, G. *et al.* Nanoscale mapping of plasmon and exciton in ZnO tetrapods coupled with Au nanoparticles. *Sci. Rep.* **6**, 19168; doi: 10.1038/srep19168 (2016).

## Supplementary Material

Supplementary Information
