# Supplementary material for: Nanoscale mapping of plasmon and exciton in ZnO tetrapods coupled with Au nanoparticles
Source: Sci Rep. 2016 Jan 12;6:19168. doi: 10.1038/srep19168 (PMC4709633; doi:10.1038/srep19168)
Supplement: Supplementary Information [file srep19168-s1.pdf]

Nanoscale mapping of plasmon and exciton in ZnO tetrapods coupled with Au nanoparticles

Giovanni Bertoni †‡, Filippo Fabbri †‡, Marco Villani †‡, Laura Lazzarini †, Stuart Turner §, Gustaaf Van Tendeloo §, Davide Calestani †, Silvija Gradečak ⊥, Andrea Zappettini †, Giancarlo Salviati †**

† CNR-IMEM, Parco Area delle Scienze 37/A, IT 43124 Parma, Italy

§ EMAT, University of Antwerp, Groenenborgerlaan 171, BE 2020 Antwerp, Belgium

⊥ Department of Materials Science and Engineering, Massachusetts Institute of Technology, Cambridge, Massachusetts (USA)

*giovanni.bertoni@imem.cnr.it; giancarlo.salviati@imem.cnr.it

Supplementary Information

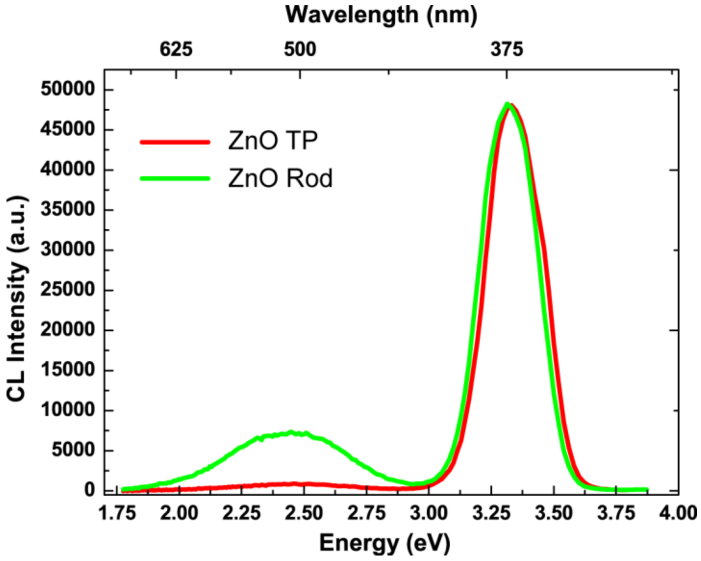

Supplementary Figure S1. Comparison between ZnO nanostructures. CL emission comparison from the ZnO nano tetrapods (red curve) synthetized here and ZnO nano rods (green curve). The common "green" emission from surface defects is severely reduced in the tetrapods, allowing to discriminate the Au plasmon (LPR) contribution in the conjugated system.

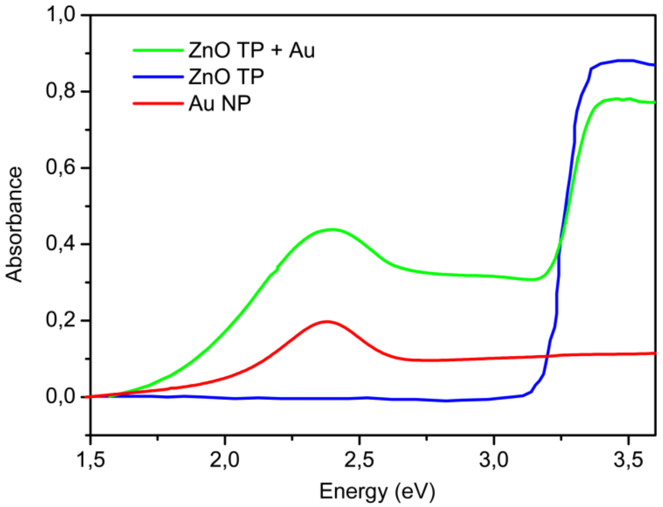

Supplementary Figure S2. Absorption spectra of the Au/ZnO nanostructures. Absorption spectra of the as-grown ZnO tetrapods showing the characteristic band-gap threshold at 3.2 eV (blue line), and

Au-functionalized ZnO tetrapods (green line). The localized plasmon resonance (LPR) is visible at 2.4 eV, while interband transitions (d $\rightarrow$ sp) are observed above 2.6 eV, as seen in the spectrum from a reference of Au NPs (red line).

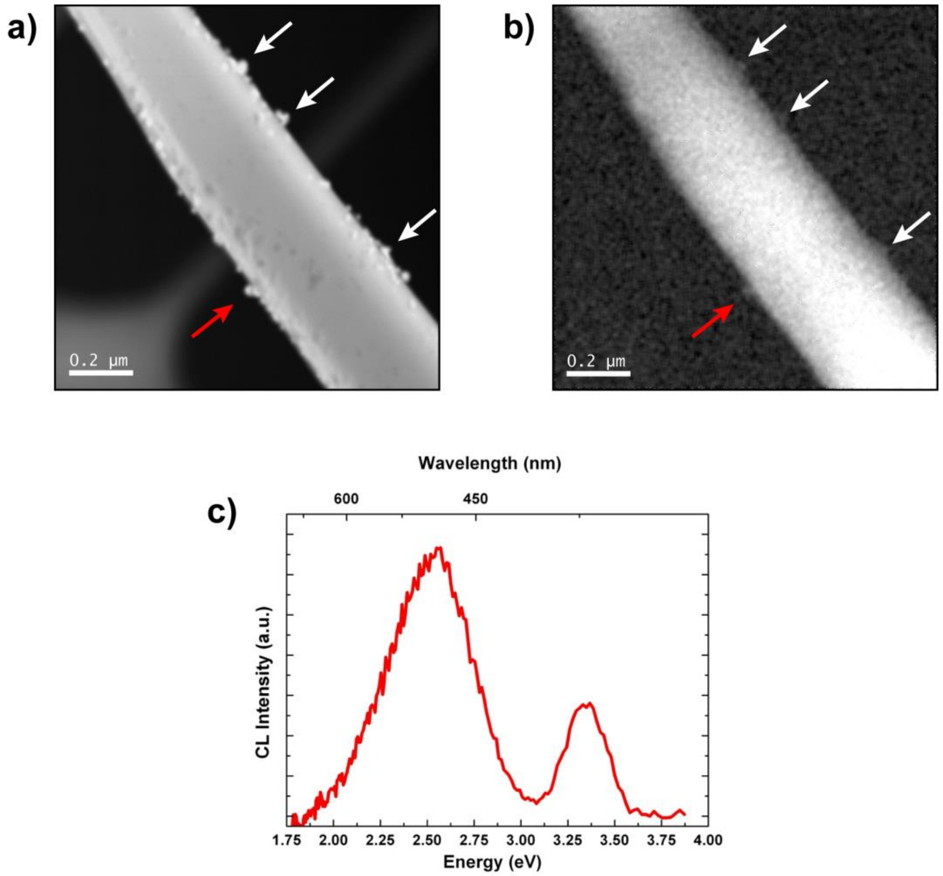

Supplementary Figure S3. Cathodoluminescence maps. a) Dark Field STEM image and b) corresponding panchromatic CL map showing emission from the Au NPs on the ZnO TP. Some NPs are indicated by the white arrows for clarity. The CL map in b) was filtered using an adaptive Wiener filter to reduce the noise in the image. c) Spot mode STEM-CL spectrum, acquired on the Au NP indicated by a red arrow in a) and b), showing the Au LPR peak as expected, and the ZnO NBE emission, indicating an optical coupling.

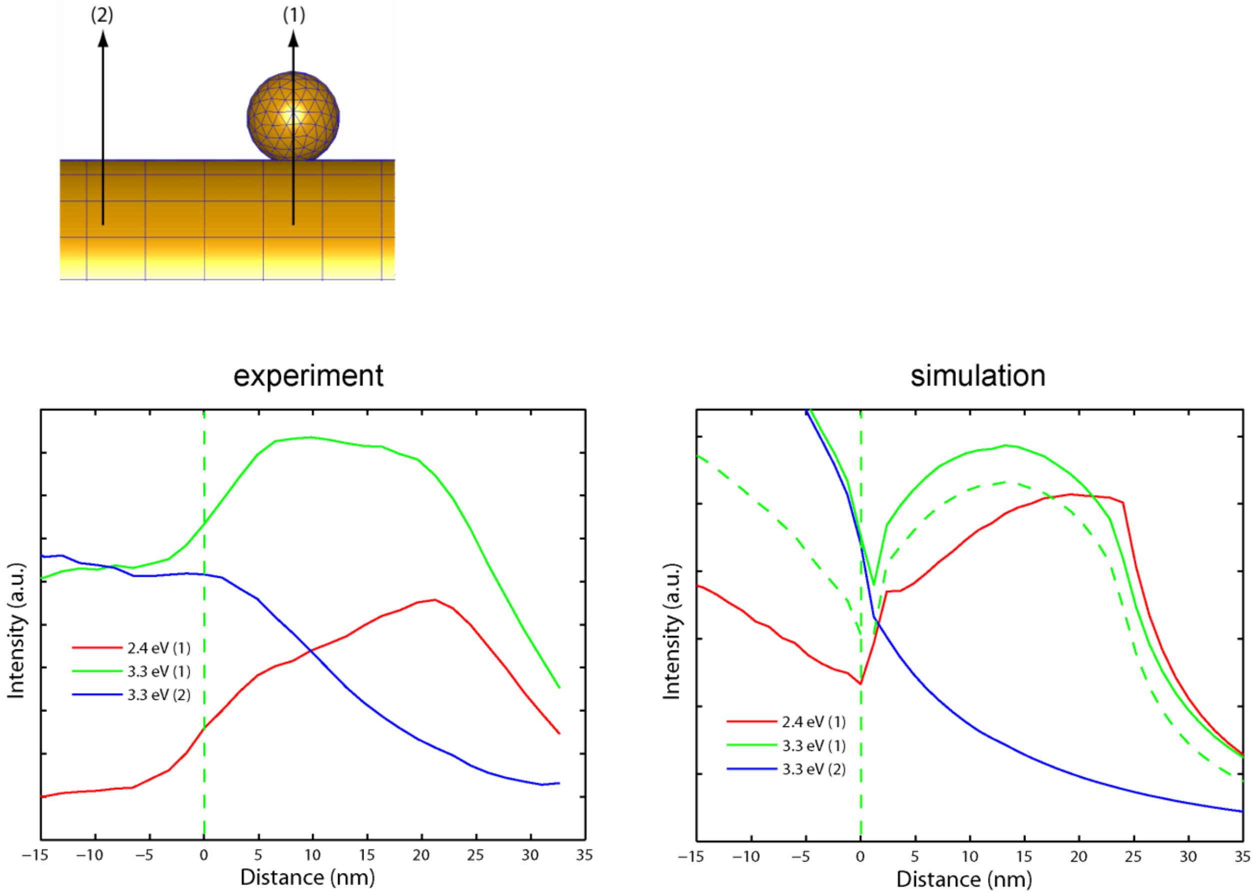

Supplementary Figure S4. Comparison between experimental and simulated profiles. Comparison of integrated linescans from the experiment (left) and the simulations (right), as extracted from the eels maps at 2.4 eV (dominated by the Au LPR) and at 3.3 eV (both Au interband and ZnO NBE contribute at this energy) without any fitting procedure. A scan across the particle (1) is compared to the scan away from the particle (2) as depicted in the sketch. In the simulation the profiles obtained from the dielectric function with the excitons (full green line) is compared to the one obtained from the dielectric function without the excitons (dashed green line). The experiment points to an extension of the ZnO excitations (both NBE and excitons) into the Au particle, with an higher contribution if excitons are taken into account.

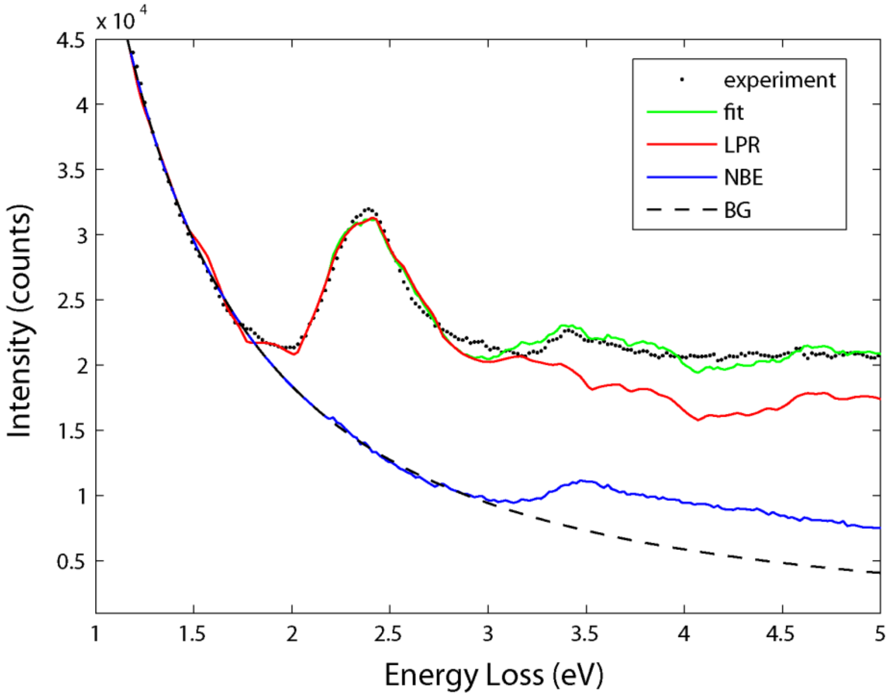

Supplementary Figure S5. Fitting model of the energy loss spectra. Example of fitting of the experimental spectrum (dotted line) from the Au/ZnO system from a pixel at the Au particle surface. The fitting resulting curve is given by the green line. The two components of the fit model used are one from Au LPR (red curve) and one from ZnO band edge (blue curve) and named NBE. The latter is taken as an approximation of the signal feature seen on the particle surface at ~3.3 eV, so to explore the spatial localization of this feature on the particle.

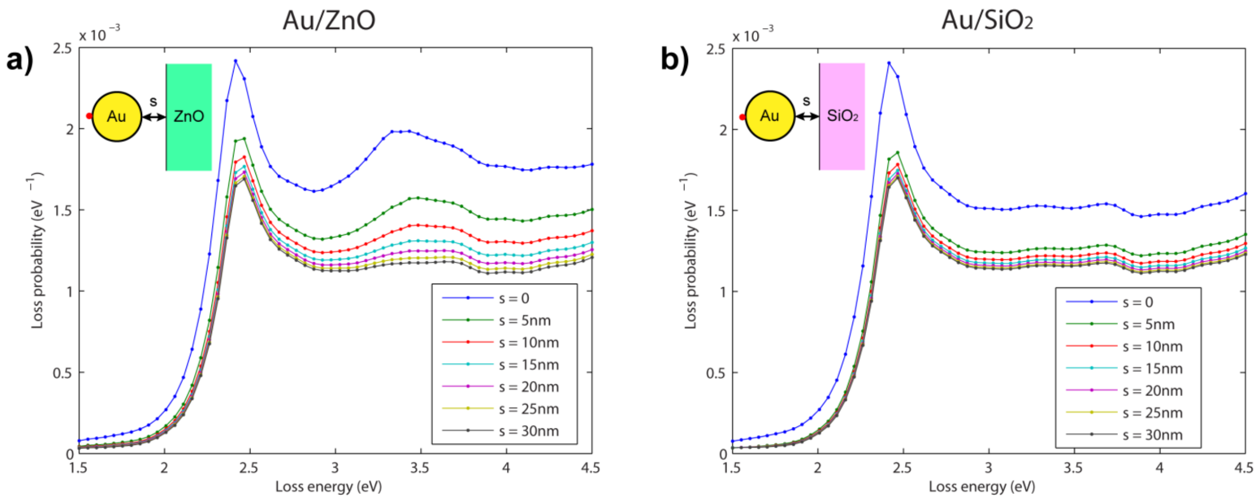

Supplementary Figure S6. Comparison between Au/ZnO and Au/SiO$_2$ nanostructures.

Comparison of the simulated energy loss probability at the Au/vacuum interface (red mark in the sketches) for the Au/ZnO case (a) and the Au/SiO$_2$ case (b). The intensity at 3.3 eV in the Au/ZnO is due to the absorption contribution from ZnO enhanced by the Au particle. In the SiO$_2$ case (transparent at 3.3 eV) only an enhancement of the overall spectral intensity at the Au/vacuum interface is notable, due to the presence of the medium (with high permittivity) on the opposite side.

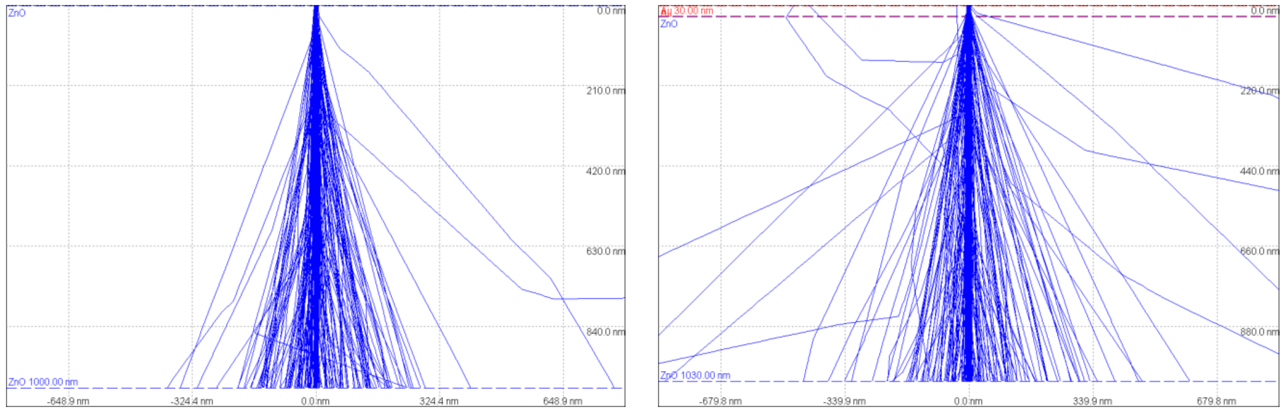

Supplementary Figure S7. Simulation of the interaction volume. Generation-recombination volume in a bare (left) and 30 nm thick gold coated (right) ZnO slab with a thickness up to 1000 nm, considering an accelerating voltage of 120 keV and a spot size of 5 nm. The Montecarlo simulations

with such accelerating voltage reveal that the electrons cross the whole materials in both the different configurations (See Fig. 1a and 1b). The main effect is a slight lateral spread of the generation/recombination volume. In addition we consider the energy release along the generation/recombination volume in both the configurations. We find that the energy release along the ZnO when coated with a 30 nm thick gold film decreases of about the 20%, but this value is strongly overestimated due to the continuous gold coating of the ZnO. In light of this effect we believe that the decrease of the ZnO emission intensity is mainly related to the gold nanoparticles absorption.
